# Structural and Electronic Properties of the Magnetic and Nonmagnetic X_0.125_Mg_0.875_B_2_ (X = Nb, Ni, Fe) Materials: A DFT/HSE06 Approach to Investigate Superconductor Behavior

**DOI:** 10.1021/acsomega.4c05894

**Published:** 2024-08-14

**Authors:** Guilherme Bonifácio Rosa, Luis Henrique da Silveira Lacerda, Sergio Ricardo de Lazaro

**Affiliations:** † State University of Ponta Grossa, Ponta Grossa, Paraná 84030-900, Brazil; ‡ Federal University of Santa Catarina, Florianópolis, Santa Catarina 88040-900, Brazil

## Abstract

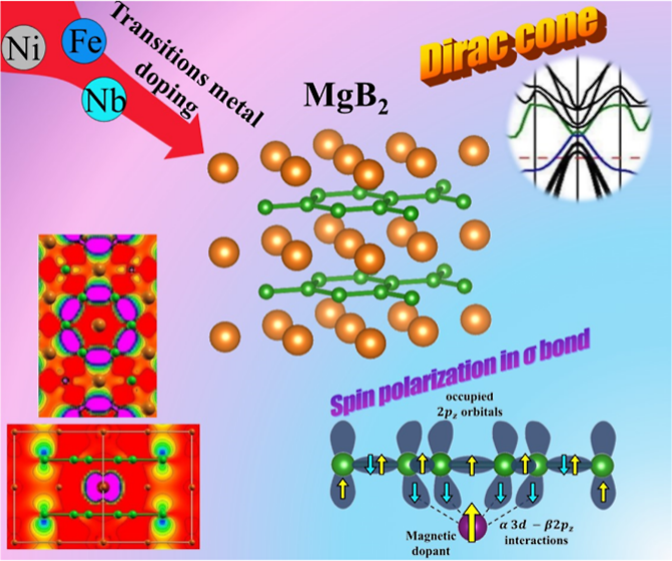

MgB_2_ material has a simple composition and structure that is well-reported and characterized. This material has been widely studied and applied in the last 20 years as a superconductor in wire devices and storage material for H in the hydride form. MgB_2_ doped with transition metals improves the superconductor behavior, such as the critical temperature (*T*_cs_) or critical current (*J*_sc_) for the superconducting state. The results obtained in this manuscript indicate that Nb-, Fe-, and Ni-doping in the Mg site leads to a contraction of the unit cell through the spin polarization on the electronic resonance of the boron layer. Fe and Ni transition metals doping perturb the electronic resonance because of stronger dopant-boron bonds. The unpaired electrons are transferred from 3d orbitals to the empty 2p_*z*_ orbitals of the boron atoms, locating α electrons in the σ bonds and β electrons in the π orbitals. The observed influence of magnetic dopants on MgB_2_ enables the proposal of an electronic mechanism to explain the spin polarization of boron hexagonal rings.

## Introduction

The climate emergency raised by energy production from fossil sources is a critical topic affecting the thermic condition of life as we know it. Alternative clean energy sources such as photovoltaics, electrolysis, tide waves, geothermics, and others are more sustainable. However, the limitation on the efficiency of these processes can be related to energy dissipation or loss on the energy generator device or energy transmission from the energy source. Such narrowness has relevant consequences for the research on advanced inorganic and organic functional materials.^1−5^ In recent years, the search for functional materials with low energy dissipation or more efficiency in the transmission process has become the focus of several scientific investigations, and advanced functional materials have been proposed to overcome this challenge.^6,7^

In this context, materials with superconducting states have been broadly investigated since their discovery. However, the application of this class of materials is limited because of the low temperatures or extremely high pressures required to maintain the superconductivity.^8−12^ The superconductor state is accessible below a given temperature, called the superconducting critical temperature (*T*_cs_), where the material transits from a normal state to a superconductor state. Since the H.K. Onnes first report on superconductors, the main challenge lies in the obtainment of materials with increased *T*_cs_, being values closer to room temperature the goal.^13,14^ A significant advantage of superconducting materials is the absence of energy loss during transmission, which is relevant to current technological purposes. Hence, this is the main property responsible for the promising expectations of the superconductor materials to surpass recent environmental problems.^15−18^ In addition, scientific or commercial devices based on superconductor materials are more efficient due to their robustness and precision.^19−21^

The magnesium diboride, MgB_2_ (MB), is an example for electronic, energetic, and data transmission devices.^22^ This material is widely investigated as a superconductor due to its *T*_cs_ near 39.0 K, a higher temperature considering the simple composition forming a crystalline structure from Mg and B layers interspersed and unusual electronic properties.^23−29^ The MB crystallizes into a *P*6/*mmm* hexagonal space group organized as multilayers with a well-defined B lattice. The Mg sites, in particular, are an excellent option to be replaced by M^2+^ transition metals, tuning the materials properties.^30^ This material is applied on bulk,^31^ thin films,^32,33^ and superconductor wires (SCW).^34−36^ The SCW under cooling leads to no loss in the energy transport process.^37^ However, if high electrical currents exceed the value of critical current, then the superconductor state is suppressed. Thus, the chemical modification of the MB was broadly reported as always connected to increased critical currents (*J*_sc_), avoiding such limitation.^38^ An usual experimental procedure to dope MB with transition metals is synthesizing them inside metallic tubes, such as Nb,^39−43^ Fe,^44,45^ and Ni^46−48^ on heat treatment to form a solid solution in Mg sites^49^. The synthesis of the MB has economic advantages regarding the YBa_2_C_u3_O_7−*x*_ (YBCO) material^50^.

Energy storage technologies are a very important electronic property in electronic transport, advanced computational devices, supercapacitors, and systems of SCW applied in superconductor coils in cryogenesis. These technologies are all based on superconductors materials, and this ensures the principal physical properties of stored charge do not decay.^51,52^

The study of MB material is attractive in materials science, especially with magnetic dopants, about the electronic nature of the boron lattice with high covalent behavior and the possibility of unpaired electron perturbation in the electronic structure of aromaticity of the B structure. The effect of unpaired electrons in superconductors is characterized in a shallow way even in current theoretical works, and this study is fundamental to understanding the electronic mechanism of magnetic impurities in high-ordered electronic structures. Lefcochilos-Fogelquist et al.^53^ reported the effect of the sp^2^ hybridization destabilization on the electronic resonance of the boron hexagonal lattice through Ti- and Ni-doping-induced the B–H hydrogenation process on the MB material. The unpaired electrons in 3d orbitals of the Ni atom were cited as a destabilization factor on sp^2^ hybridization, as observed on density of states (DOS) analysis. The strong bond between the transition metal and B atoms hinders the B–H bond formation; therefore, the hydrogenation process is not efficient. Wan and coauthors^54^ evaluated the MB as anodes for application in next-generation Li-ion batteries through DFT/PBE in the VASP code. The results discussed indicate the potential of MB based on the enhanced electronic resistivity and transport and low cost compared with other commercialized anodes.

The present study shows a DFT/HSE06 investigation on the electronic changes of the Nb-, Ni-, and Fe-doping in the Mg sites of the MB structure to clarify the role of the sp^2^-hybridized orbitals of the boron layers on the superconducting state.^55−59^

## Computational Methodology

### Structural Description for the MB and XMB Models

**Figure 1:**
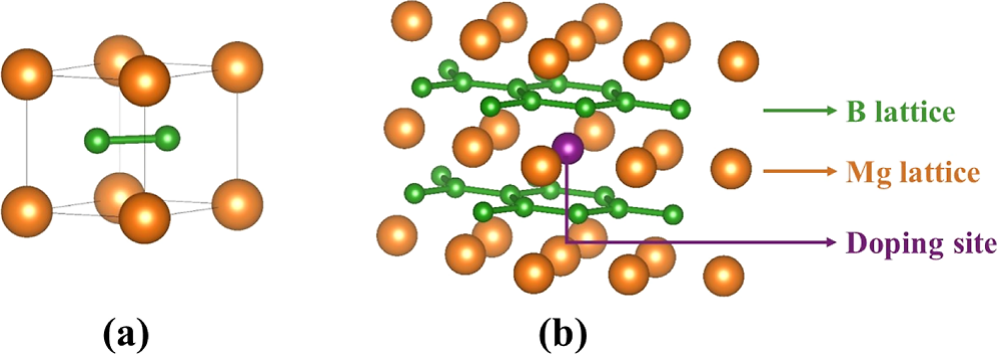
Crystalline representation in the *P*6/*mmm* group of the periodic models for MB (a) and XMB (X = Ni, Fe, Nb) (b) from the DFT/HSE06 approach. The orange, green, and purple colors represent Mg, B, and X-dopant atoms, respectively.

A periodic structural model for the lamellar MB was built from experimental data for the *P*6/*mmm* hexagonal group,^60^ yielding to MB (Figure 1a) and X_0.125_Mg_0.875_B_2_ (X = Nb, Ni, Fe) (XMB) materials (Figure 1b). The XMB models are a 2 × 2 × 2 bulk expansion, in other words, a growth of 2 cells in a, b, and c lattice parameters resulting in 8 cells to guarantee a X-doping concentration of 12.5% into the Mg site. Such proposed models include nonmagnetic and magnetic systems, with spin-polarized calculations required for the latter type. Thus, Nb_0.125_Mg_0.875_B_2_ (NbMB) are treated from closed-shell DFT calculations. At the same time, Ni_0.125_Mg_0.875_B_2_ (NiMB) and Fe_0.125_Mg_0.875_B_2_ (FeMB) were evaluated from restricted open-shell calculations assuming a collinear method to represent the magnetic ordering, describing the unpaired electrons as symmetrically distributed along the Cartesian coordinates (*x*, *y*, *z*).

### Chemical Bond Energy Calculation

The formation energies of the MB (*E*_fMB_) and XMB materials (*E*_fX_) were calculated from eqs 1 and 2, where *E*_B_ is the energy of the boron atom, *E*_Mg_ is the energy of the magnesium atom, and *E*_X_ is the energy of the doping atom. Meanwhile, eq 3 presents the formalism for calculating the X-doping bond energies (*E*_bondX_) in the XMB materials; factor 1/6 is the energy correction regarding the six chemical bonds found on the doping site. In eq 3, the binding energy (*E*_bind_) is the necessary energy to break chemical bonds of the X dopants with positive values.
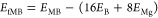
1
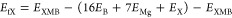
2
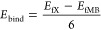
3

### Properties Analysis

Band structure (BS), DOS, and spin charge density maps (SCDM) analyses were performed from the wave functions for the fully relaxed MB and XMB materials. In particular, the band gap region of the materials was evaluated since the DOS and BS calculations take into account the last five energy levels of the valence band (VB) and the first five states of the conduction band (CB) and determine the data for the BS and DOS analysis. In the case of charge density maps, SCDM assesses the electronic density only for unpaired electrons in alpha and beta spins; therefore, the spin density maps for nonmagnetic NbMB and MB were not calculated. Such analysis carefully depicted the charge distribution in the dopant transition metal and the boron atoms within the boron layers since the (001) and (110) planes were investigated. VESTA^61^ and XCrysden^62^ packages were employed in this step.

### Input Information

The three-dimensional periodic DFT quantum simulations performed in this study describe the exchange–correlation energies through the formalism proposed in the Heyd−Scuseria−Ernzerhof functional (HSE06)^63−65^ implemented in the CRYSTAL17 code.^66^ All simulations assume the following thermodynamic conditions: gas phase, zero Kelvin, and vacuum on a stationary position approximation. Mg, B, Fe, Ni, and Nb atoms were described by the 8−511G,^67^ m-6-311G(d),^68^ s86411p6411d4411,^69^ 84−2111(6311d)(4111d)G,^70^ and 986-31(631)G^71^ Gaussian basis sets, respectively. A 8 × 8 × 8 point mesh filled the space on the symmetry *k*-points for the BS described by the Monkhrost–Pack approach.^72^ The self-consistent field convergence criterium was of 10^−7^ a.u., the total energy convergence for the structural relaxation was 10^−8^ a.u., and the factor of 10^−8^ a.u. in energy truncated the convergence of the mono- and bielectronic integrals.

## Results and Discussion

### Structural and Charge Density Analysis

Quantum simulation of the MB material calculated *a* = *b* = 3.073 Å and *c* = 3.553 Å lattice parameters, which agree with previous experimental^73^ and theoretical studies found in scientific literature.^74,75^ Thus, the obtained results show the efficiency of the employed methodology in predicting the material crystalline structure.

As expected, Fe-, Ni-, and Mg-doping is responsible for crystalline structural distortions of the MB material (Table 1). The NbMB showed an expansion of 0.016 Å in the *a* and *b* lattice parameters and a 0.018 Å contraction in the *c* lattice parameter. The unit cell angles indicate a low distortion of 0.002° in the γ angle. In FeMB, a contraction of 0.015 Å in the *a* and *b* lattice parameters and 0.033 Å for the *c* parameters without modifications on unit cell angles. The results for NiMB suggest a contraction of all lattice parameters, with the most pronounced decrease observed for the c lattice parameters; the angles were also affected. The modifications carried out from 3d (Ni and Fe) and 4d (Nb) metals in the Mg site preserved the *P*6/*mmm* crystalline structure since they are responsible only for point distortions, which affect the lattice parameters and unit cell angles. The chemical bonds directly influenced the volume of the cell unit, decreasing the volume following the order MB > NbMB > FeMB > NiMB.

**Table 1:** Lattice Parameters, Volume, and Lattice Angles for the MB, NbMB, FeMB, and NiMB Materials

material	*a* (Å)	*b* (Å)	*c* (Å)	volume (Å^3^)	α = β	γ
MgB_2_ exp^73^	3.073	3.073	3.553	29.057	90.0°	120.0°
MgB_2_	3.067	3.067	3.436	27.985	90.0°	120.0°
Nb_0.125_Mg_0.875_B_2_	3.083	3.062	3.418	27.774	90.0°	120.002°
Fe_0.125_Mg_0.875_B_2_	3.052	3.052	3.403	27.448	90.0°	120.0°
Ni_0.125_Mg_0.875_B_2_	3.055	3.055	3.388	27.371	90.0°	120.068°

**Figure 2:**
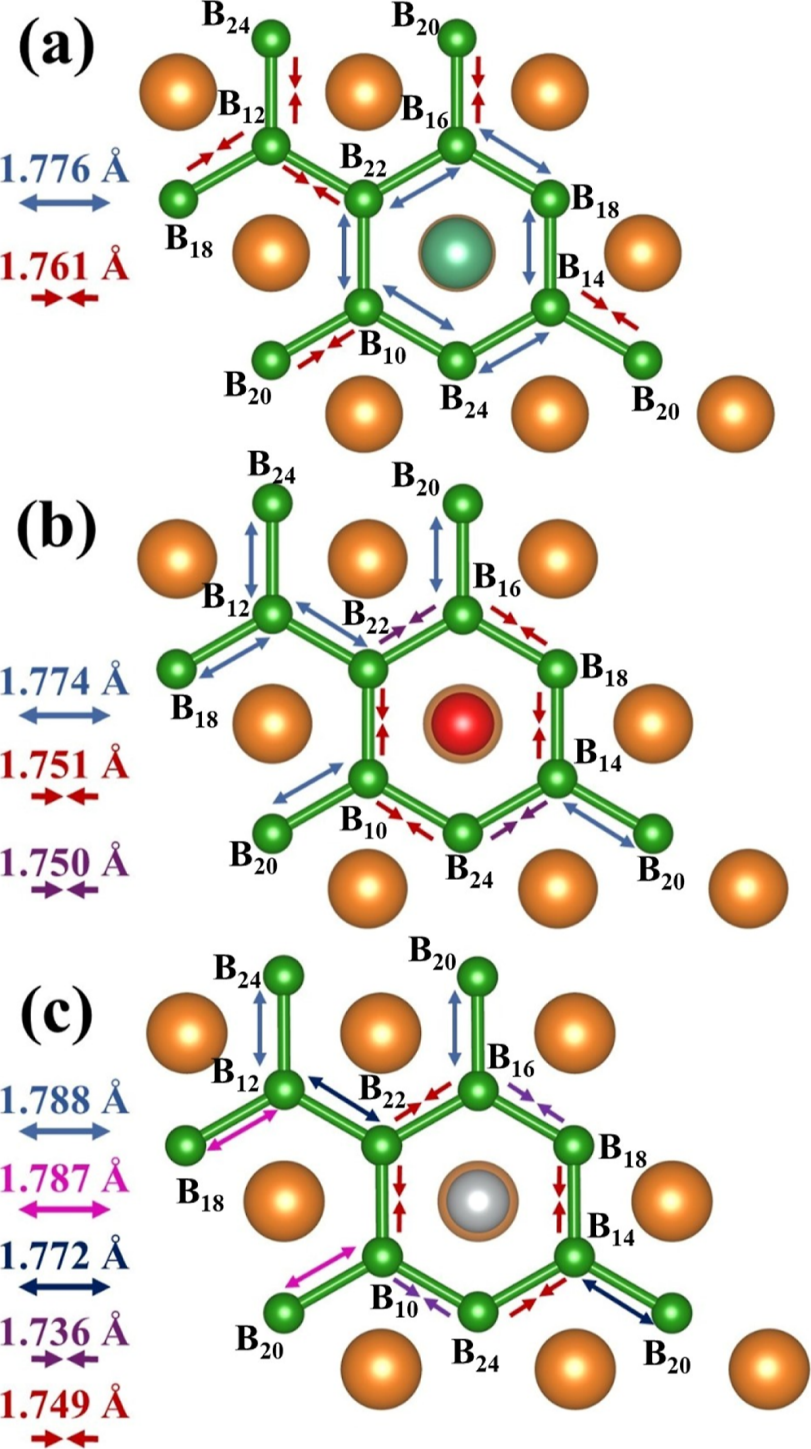
Chemical bonds for the boron layers in NbMB (a), FeMB (b), and NiMB (c) materials indicating expansion and contraction effects. The orange, green, cyan, red, and silver colors represent Mg, B, Nb, Fe, and Ni atoms, respectively. The blue arrows represent an expansion of the B–B bond length, while the red arrows represent a contraction in the B–B bond length.

The results indicate that Nb-, Fe-, and Ni-doping decreases the distance between the B and cation layers, distorting the c lattice parameter according to the X transition metals. The calculated distances are 2.467 Å (B, Mg), 2.453 Å (B, Nb), 2.429 Å (B, Fe), and 2.430 Å (B–Ni). Figure 2 shows the B–B bonds for the XMB materials. The B–B bond length within the boron layer is 1.770 Å for the MB material. After Fe, Ni, and Nb-doping, slight distortions created expansion and contraction effects on the B hexagons (Figure 2). Consequently, such distortions perturbed the electronic resonance on the boron layers.

**Figure 3:**
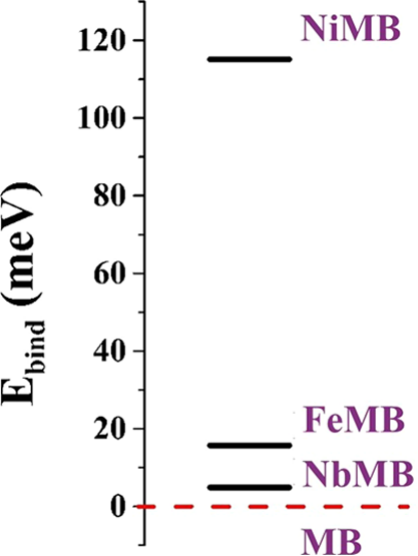
Calculated bond energies for the X-doped (X = Nb, Fe, Ni) MgB_2_ structure. The scale was referenced to the Mg–B bond energy in MgB_2_ material, represented by the red dash line at 0 eV.

Formation energy is relative to an enthalpy connected to electronic energy under drastic thermodynamic conditions from the stationary state approximation accepted in quantum simulations. Such an approximation package determines good qualitative representativity for chemical properties, such as the chemical bond energies. The X–B and Mg–B bond energies were calculated to understand the interaction between boron and cations layers. The results presented regarding the Mg–B bond energy in the MB material (Figure 3) showed that the Ni–B and Fe–B bonds are more robust, corroborated by Figure 2. Consequently, the Fe- and Ni-doped materials showed more significant distortion of the boron ring in the direction of the dopant site. This structural effect indicates that Ni–B and Fe–B bonds are more effective than Nb–B and Mg–B bonds. For the MB, the Mg–B bond presents a weak covalent character because of the null overlap between Mg 2s orbitals and empty 2p_*z*_ orbitals of the boron, causing charge density wave behavior for the electrons on the boron layers.

The NbMB material presents a low bond energy of 4.9 meV, which is very similar to that observed for the Mg–B bond in pure material. The Fe- and Ni-doping increased the bond energy to 15.7 and 115.1 meV because of the unpaired electrons. This behavior justifies a more efficient overlap between the 3d orbitals of the transition metals and the 2p_*z*_ orbitals of the boron layer.

The spin populations for the FeMB and NiMB materials are listed in Table 2, showing significant differences for each material. The total magnetic moment (μ_total_) of the FeMB material is 2.539 |e^−^| in the alpha orientation on the Fe 3d orbitals. However, the Fe atomic spin moment (μ_Fe_) is 3.094 |e^−^| in alpha, while the spin moments on the boron atoms (μ_B_) are 0.576 alpha and 1.092 beta |e^−^|. The boron spin moments with Fe and Mg atoms were μ_B–Fe_ = 0.091 beta |e^−^| and μ_B–Mg_ = 0.144 alpha |e^−^|. In NiMB, the μ_total_ is precisely 2.0 |e^−^| with the alpha spin ion, which is 1.244 alpha |e^−^| on the Ni atoms and 0.712 alpha electrons in B atoms. The simulation does not present a significant beta electron population on boron atoms. In different sites of boron only, there are magnetic moments for alpha electrons, while the μ_B–Mg_ is higher than μ_B–Ni_. The same tendency occurs for the FeMB material. Then, it is possible to classify two different boron sites, i.e., boron bonded directly with dopant atoms (B–Fe and B–Ni) and boron without direct bond with such dopants (B–Mg). Consequently, there is a different spin population for each B–Fe, B–Ni B, and B–Mg sites because of the chemical environment in the boron lattice.

**Table 2:** Results for the Total Spin Momentum (μ_total_) for FeMB and NiMB^a^

FeMB		NiMB	
μ_total_	2.539^α^	μ_total_	2.000^α^
μ_Fe_	3.094^α^	μ_Ni_	1.244^α^
μ_B_	0.576^α^	μ_B_	0.712^α^
	1.092^β^		0.0^β^
μ_B–Fe_	0.091^β^	μ_B–Ni_	0.020^α^
μ_B–Mg_	0.144^α^	μ_B–Mg_	0.120^α^

a Atomic spin moment for the Fe atom (μ_Fe_) on B atom (μ_B_); B atoms bonded with magnetic dopants (μ_B–Fe_, μ_B–Ni_), and B atoms connected to the Mg atoms (μ_B–Mg_).

**Figure 4:**
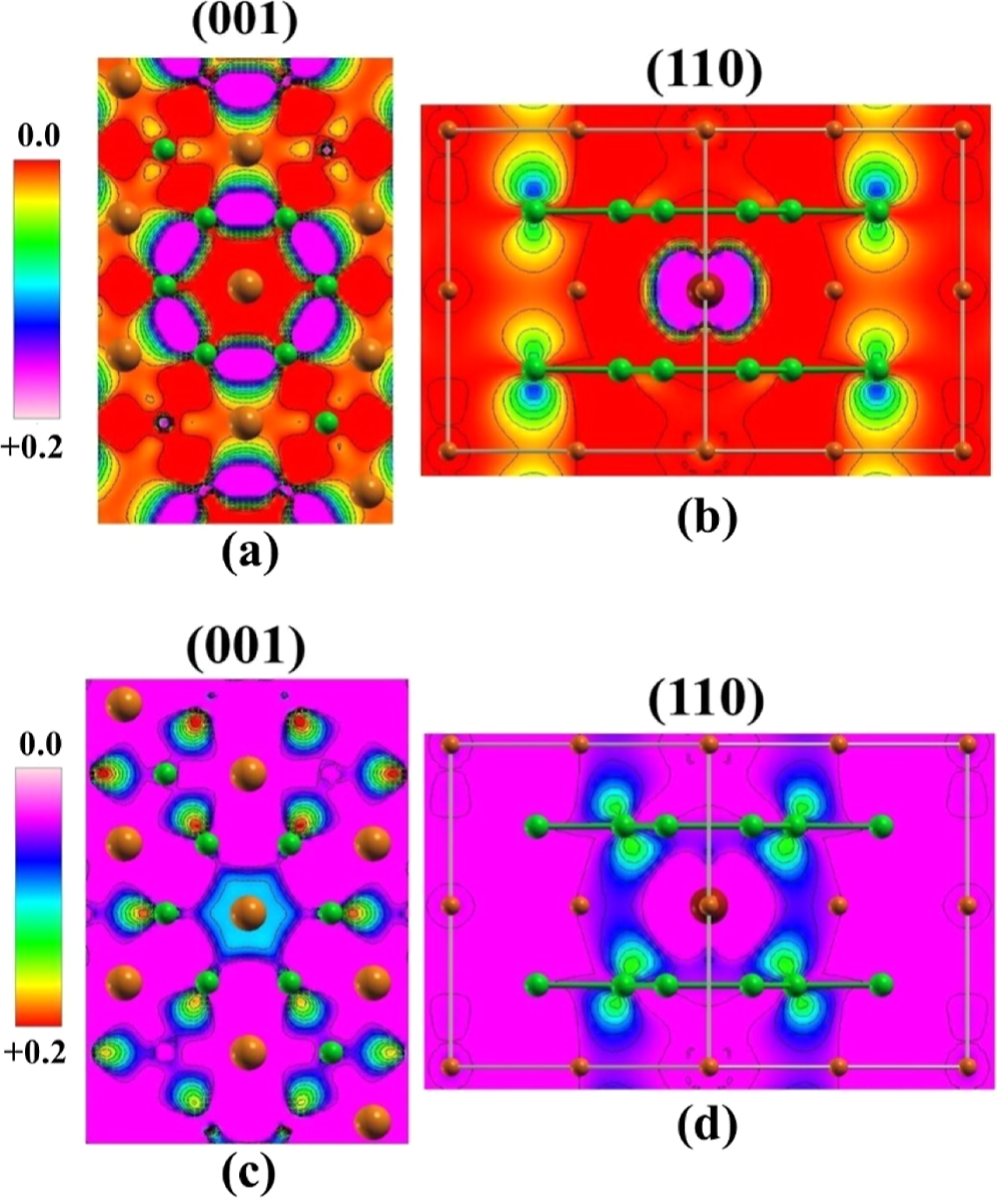
SCDM for the Fe_0.125_Mg_0.875_B_2_ material in the electronic channels (a) alpha channel in the (001) plane; (b) alpha channel in the (110) direction; (c) beta channel in the (001) plane; and (d) beta channel in the (110) plane. The green, orange, and red colors represent the B, Mg, and Fe atoms, respectively.

Figure 4 shows the SCDM for the FeMB magnetic system, demonstrating that the boron lattice presents alpha spin density on the 2p_*x*_ orbitals involved in the formation of the boron ring found in the (001) plane (Figure 4a). The evaluation of the beta channel for the same plane (Figure 4c) suggests that such unpaired electrons occupy the B 2p_*y*_ orbitals, which connect two different boron rings. In the case of (110) planes, the unpaired electron occupation depends on the B distance to the Fe dopant, being alpha electrons found in the 2p_*z*_ orbitals of the furthest sites and beta electrons observed at the B 2p_*z*_ orbitals for the sites close to the doping site. It is important to note that the orbitals containing the beta electrons are strongly influenced by the Fe atoms, resulting in 2p_*z*_ orbitals aligned in the direction of the inserted transition metal. The spin canting creates a deformation on the spin density, which is found in the internal and peripheral regions of the boron rings (Figure 4c), indicating that unpaired electrons have an anisotropic magnetic effect on the B 2p_*z*_ orbital in both alpha and beta channels.

An important detail in FeMB spin-charge density maps is the measure of the spin canting on the p_*z*_ orbitals with beta electrons. The spin canting for the B 2p_*z*_ orbital oriented to the Fe atom has a torsion of 35°, while the same orbital oriented to the Mg atom shows 22° of torsion (Figure 4d). The result indicates a charge attractor behavior of the Fe dopant in the material structure, behavior coherent with the structural distortions on the boron ring in Figure 2b.

The Fe atoms are responsible for the magnetic moment created within the boron hexagonal rings, affecting the electronic resonance in the B–B bond. In summary, Fe atoms within the MgB_2_ structure are responsible for the change of 2p orbital energies of all boron, creating a magnetic moment for such species, as evidenced by the spin-charge values in Table 2. More specifically, the unpaired electrons in 2p_*z*_ orbitals of the boron lattice present an increased overlap with the Fe atoms, leading to a stronger Fe–B bond regarding the Mg–B bond.

**Figure 5:**
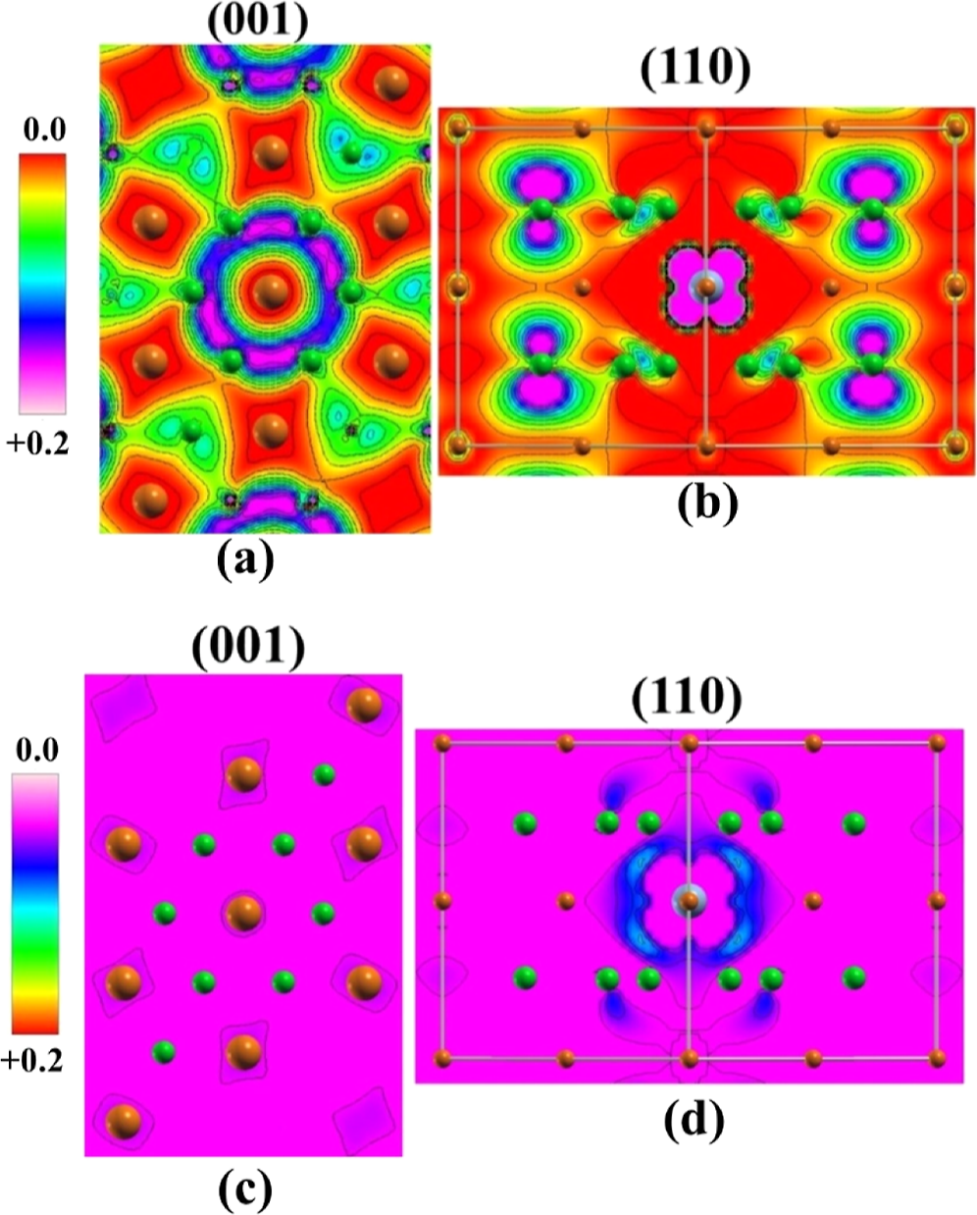
Spin Charge Density Map of the Ni_0.125_Mg_0.875_B_2_ material in (a) alpha channel in the (001) plane; (b) alpha channel in the (110) direction; (c) beta channel in the (001) plane and (d) beta channel in the (110) plane. in electronic channels. The colors green, orange, and red represent the B, Mg, and Fe atoms, respectively.

For NiMB, the alpha electrons show a low spin density located in the boron ring bonds in the (001) plane (Figure 5a). Meanwhile, for the (110) plane, there is a high spin density on the 2 p_*z*_ orbital of the boron. The difference between NiMB and FeMB can be attributed to the characteristic spin moments for unpaired electrons in the d orbitals of the Ni (1.244 |e^−^|) and Fe (3.094 |e^−^|) atoms. NiMB spin density is less ordered regarding FeMB material. This fact is evident in the (001) plane of material in B–B bonds, in which alpha electrons present a distorted spin density (Figure 5b). Such electronic behavior is a consequence of different magnetic moments between Fe and Ni atoms. The Fe atom presents 3.094 |e^−^| alpha in 3d orbitals, while Ni presents 1.244 |e^−^| alpha in 3d orbitals. The strong magnetic moment on the Fe atom provides a more spin distribution of the alpha electrons in B–B bonds and beta electrons in the 2 p_*z*_ orbital of the boron atoms. NiMB material does not present beta unpaired electrons in the electronic structure, indicating that the Ni unpaired electrons are insufficient to provide an effective spin polarization on the boron layer in the MB material.

### Electronic Profile

**Figure 6:**
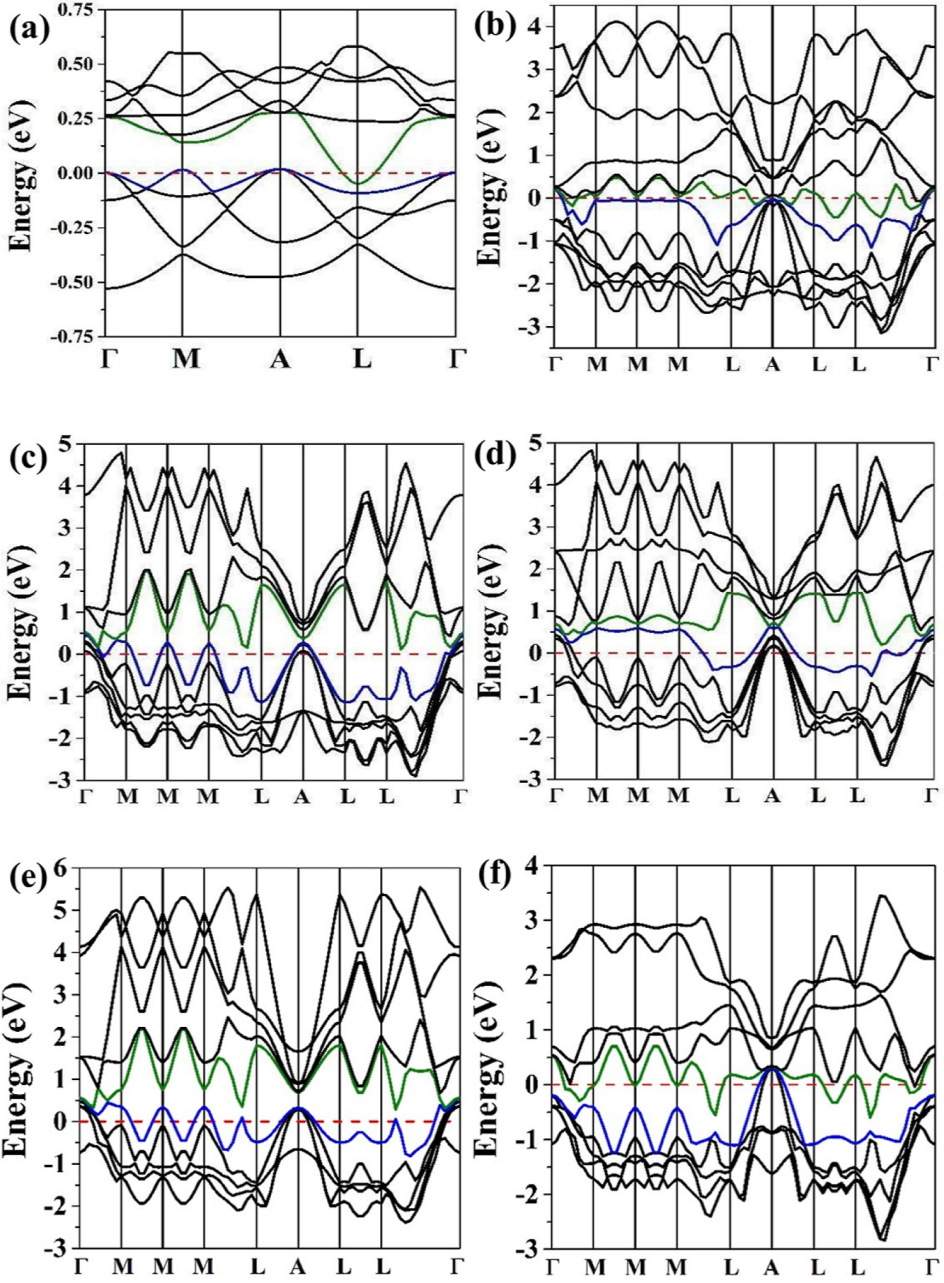
Band structures for MB (a), NbMB (b), alpha FeMB (c), beta FeMB (d), alpha NiMB (e), and beta NiMB (f). The blue lines represent the last energy level on the VB, while the green lines represent the first energy level of the CB. Red dot lines refer to the Fermi level referenced in 0 eV. The coordinates for the *k* points in BS analysis were Γ(0,0,0), *M*(1/2,0,0), *M*(0, 1/2,0), *M*(1/2, 1/2, 0), *L*(1/2, 0, 1/2), *A*(0, 0, 1/2), *L*(0, 1/2, 1/2), *L*(1/2, 1/2, 1/2).

The DOS and BS electronic properties evaluated the MB and XMB materials. The BS for the MB (Figure 6a) presented a metallic behavior in direct bandgaps of 0.05 eV at the *L* point and 0.12 eV at the M point. The NbMB (Figure 6b) is a nonmagnetic material exhibiting a metallic profile structure with Dirac cones located explicitly at the Γ and *A* points. The FeMB and NiMB materials have unpaired electrons displacing the electronic bands in the alpha and beta states. Figure 6c,d shows the BS for FeMB where Dirac-cones are at the Γ point in both alpha and beta spins. On the other hand, NiMB (Figure 6e,f) indicates two Dirac cones at the Γ point of the alpha spin and one cone at the *A* high-symmetry point of the beta channel. It is essential to highlight that doping causes punctual defects responsible for the repetition of the *M* and *L* high-symmetry points in the electronic structure of NbMB, FeMB, and NiMB.

**Figure 7:**
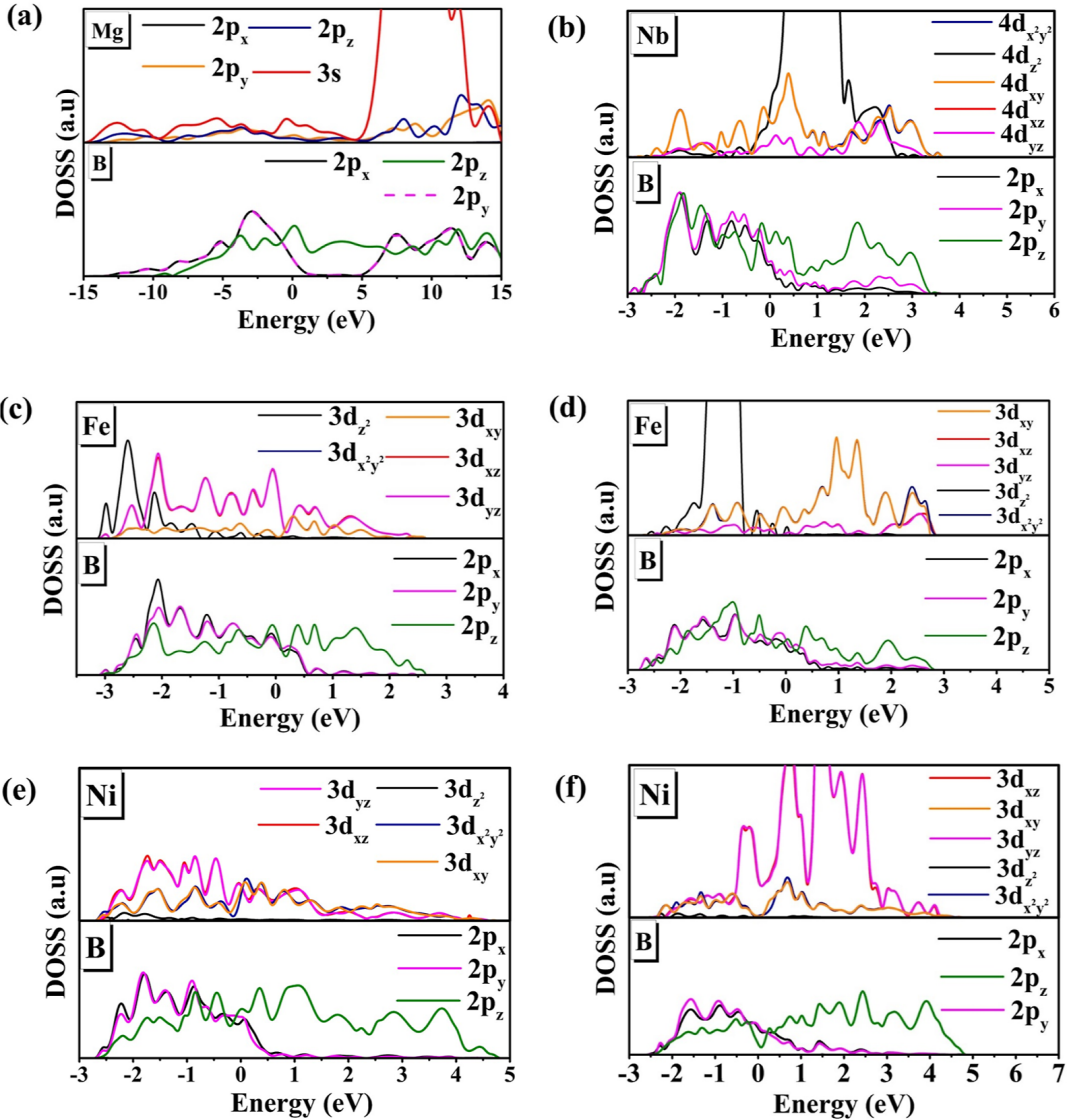
DOS per orbital for MB (a), NbMB (b), FeMB alpha channel (c), FeMB beta channel (d), NiMB alpha channel (e), and NiMB beta channel (f).

The electronic behavior reported for materials based on the MB structure presents the Mg atoms as electron donors to the boron atoms. The B layers have σ-bond features from p_*x*_ and p_*y*_ orbitals and π-bond features in the p_*z*_ orbitals.^76^ Then, the orbital DOS analysis (Figure 7) demonstrates the contributions of the atomic orbitals, specifying the overlap among B 2p orbitals, the 2p and 3s orbitals from Mg, the 4d orbitals from Nb, and the 3d orbitals from Fe and Ni atoms. In Figure 7a, the DOS for the MB material shows that the p_*x*_ and p_*y*_ orbitals for boron are degenerated, while the p_*z*_ orbital contribution is found mainly at 0 eV.

In other words, the 2p_*z*_ orbitals are predominant in the Fermi energy region, being overlapped with the 2p and 3s orbitals of the Mg atom; such energy levels create an interaction between the Mg and B layers. The boron layer in the NbMB material (Figure 7b) interacts with Nb through linear (2p_*z*_−4d_*z*_^2^) or parallel ( and 2p_*z*_–4d_*xy*_) orbitals. The 4d_*z*^2^_ orbital Nb presents a high electronic state density on the top of the VB and bottom of the CB overlapping with the 2p_*z*_ orbitals from boron. In turn, the 4d_*xz*_ and 4d_*yz*_ orbitals have a small contribution to the formation of the VB and CB.

For the FeMB (Figure 7c,d), the behavior is opposite regarding the NbMB material. The Fe atoms present distinct 3d−2p overlap in alpha and beta channels. In the alpha spin channel (Figure 7c), there is an overlap between 3d (3d_*xz*_ and 3d_*yz*_) orbitals Fe and 2p (2p_*x*_ and 2p_*y*_) orbitals boron. Meanwhile, in the beta channel, overlap occurs between the 3d_*z*^2^_ and 2p_*z*_ orbitals. These results corroborate the SCDM analysis since σ-bonding orbitals were found in the alpha channel (Figure 4a) and π-bonding orbitals are observed in the boron layer (Figure 4b). In the case of NiMB, the orbital overlap is less pronounced than in FeMB. The alpha and beta channels in Figure 7e show the overlap between Ni (3d_*xz*_ and 3d_*yz*_) and boron (2p_*x*_ and 2p_*y*_) orbitals. The lack of overlap between 3d_*z*^2^_ orbital Ni and 2p_*z*_ orbital boron is the reason behind the significant spin charge density in the alpha channel (Figure 4a) and the spin absence in the beta channel (Figure 4b) for the NiMB.

**Figure 8:**
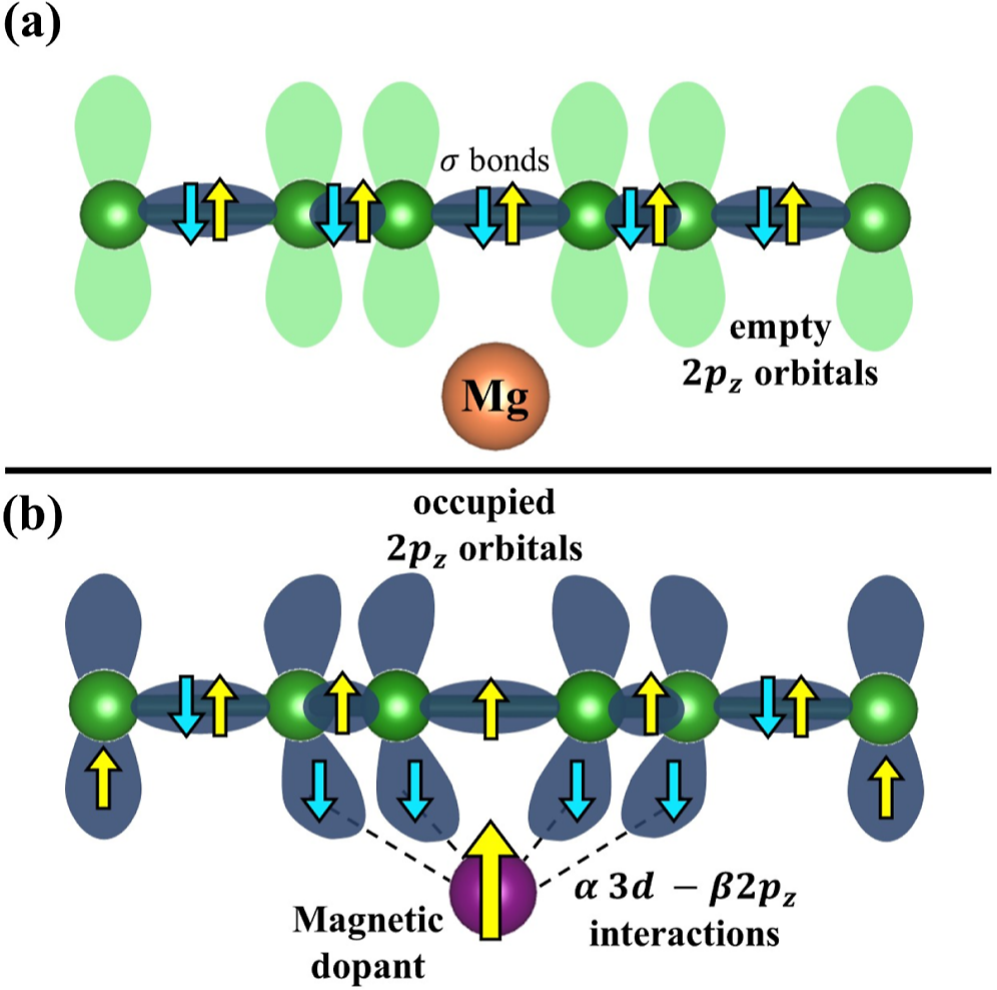
Mechanism of spin polarization for the FeMB and NiMB. Green and purple spheres refer to the boron ring and magnetic dopants. The red and blue arrows represent the alpha and beta spins, respectively.

The results enable us to better understand the influence of magnetic dopants on π resonance in the boron layers, creating spin polarization. The high spin moment localized in the dopant site is sufficient to perturb the p_*x*_ and p_*y*_ overlap responsible for forming the boron layer. As previously mentioned, the perturbation of electron resonance occurs only for magnetic dopants, the magnitude of the spin polarization being proportional to the impurity magnetic moment. Therefore, the magnetic moment uncouples the alpha and beta spins in σ-bond B–B, locating alpha electrons in the σ-bond region and β electrons in π orbitals, creating the electronic coupling between α2p_*z*_–β3d orbitals (Figure 8). In the investigated materials, the Ni atom has two unpaired electrons, while the Fe atom presents four unpaired electrons. The SCDM (Figure 5) indicated that only alpha-unpaired electrons are in the boron lattice of the NiMB. In contrast, alpha and beta electronic densities are well-defined in the boron lattice in FeMB. Thereby, the high spin moment is essential to locate the unpaired electrons on the empty 2p_*z*_ orbitals in the boron layer. This observation is coherent with previous theoretical results, which determine the electronic effect of the Fe- and Ni-doping on the MB material, suggesting that the boron atom presents a stronger chemical bond with the magnetic atom than the nonmagnetic atom.^53^

Based on the destabilization of the B–B bond caused by the presence of the magnetic transition metal and the M–B bond energies analysis (Figure 3), an electronic mechanism for spin polarization was proposed (Figure 8). The mechanism agrees with other ideas previously reported in the literature, contributing to clarifying the decrease in *J*_c_ and *T*_c_ values from Fe and Ni doping in MB materials. In other words, the doped materials lose superconductor efficiency because the localization of the alpha and beta electrons harms the cooper pair formation.^44,53,77^ In addition to the spin polarization effect, an electronic transfer process is observed since the Fe atom transfers 3d_*z*_ electrons to empty 2p_*z*_ orbitals in the boron atoms, making a strong chemical bond. Magnetic dopants behave as quantum charge attractors in MB structures, with a selection to attract the beta electrons from the boron layer electronic resonance and pair them with alpha electrons in the 3d orbitals.

The Spin Polarization Mechanism is theoretical evidence to explain the loss of superconductivity efficiency of the FeMB and NiMB reported in experimental works. The break of electronic pairing in the boron layer makes Cooper pair formation difficult. Furthermore, magnetic dopants create magnetic centers with the behavior of quantum charge attractors because they separate electrons per spin in different orbitals of boron atoms.

## Conclusions

The present work investigates the influence of Fe-, Ni-, and Nb-doping on the properties of MgB_2_. The results obtained for the MgB_2_ material, with a crystalline structure that aligns with experimental reports, demonstrate the potential and promise of the employed theoretical methodology. The doping with 3d and 4d transition metals induces a contraction of the crystalline structure, maintaining the *P*6/*mmm* space group. The electronic structure results suggest that the superconductivity in doped MgB_2_ materials can potentially offer a more accessible superconducting state due to the presence of Dirac cones in the Γ and *A* points. Therefore, modifying MgB_2_ materials with the investigated dopant species is a promising alternative for various applications, sparking excitement and anticipation for future possibilities in the field.

In particular, Fe- and Ni-doping showed structural and electronic modifications connected to the unpaired electron population in the 3d orbitals, promoting an effective bond with the boron layer. The results enable the proposal of an electronic mechanism for spin polarization of boron layers through the unpaired electron transfer from the Fe atom, causing a perturbation of π resonance on boron rings. Furthermore, the magnetic doping in the MgB_2_ material increases chemical bonds between boron atoms and transition metal dopants because of the spin polarization.

The Spin Polarization Mechanism presented a strong spin separation on the boron ring from magnetic induction of the Fe atoms.
